# Primary Testicular Non-Hodgkin’s Lymphoma With Bilateral Adrenal Metastasis: A Rare Presentation

**DOI:** 10.7759/cureus.43766

**Published:** 2023-08-19

**Authors:** Sanket Mane, Suresh Phatak, Avinash Dhok, Azhar S Shaikh

**Affiliations:** 1 Radiodiagnosis, NKP Salve Institute of Medical Sciences and Lata Mangeshkar Hospital, Nagpur, IND

**Keywords:** computed tomography of scrotum, ultrasound scrotum, non-hodgkins lymphoma, adrenal metastasis, testicular lymphoma

## Abstract

Primary testicular lymphoma is the common testicular neoplasm in patients aged more than 65 years. It accounts for a small number of cases of adult testicular malignancies. Though the metastasis to bone marrow, liver, and central nervous system are well known, metastasis to adrenal glands is a very rare entity. It can be mistaken as a germ cell tumor or a dual malignancy. To rule out other causes, a multidisciplinary approach is required. Here, we present a rare case of primary testicular Non-Hodgkin’s lymphoma with bilateral adrenal metastasis.

## Introduction

Primary testicular lymphoma constitutes merely 5% of all adult testicular malignancies and is an uncommon form of cancer. Among men aged 50 years and above, it is the predominant type of testicular malignancy [1]. It is an aggressive lymphoma and constitutes only 1-2% of all non-Hodgkin’s lymphomas [2]. Diffuse large B-cell lymphoma represents the most common subtype among primary testicular lymphoma [3]. Metastasis of testicular lymphoma is common in the contralateral testis, brain, lung, peritoneum, omentum, and bone [4].

## Case presentation

**Figure 1:**
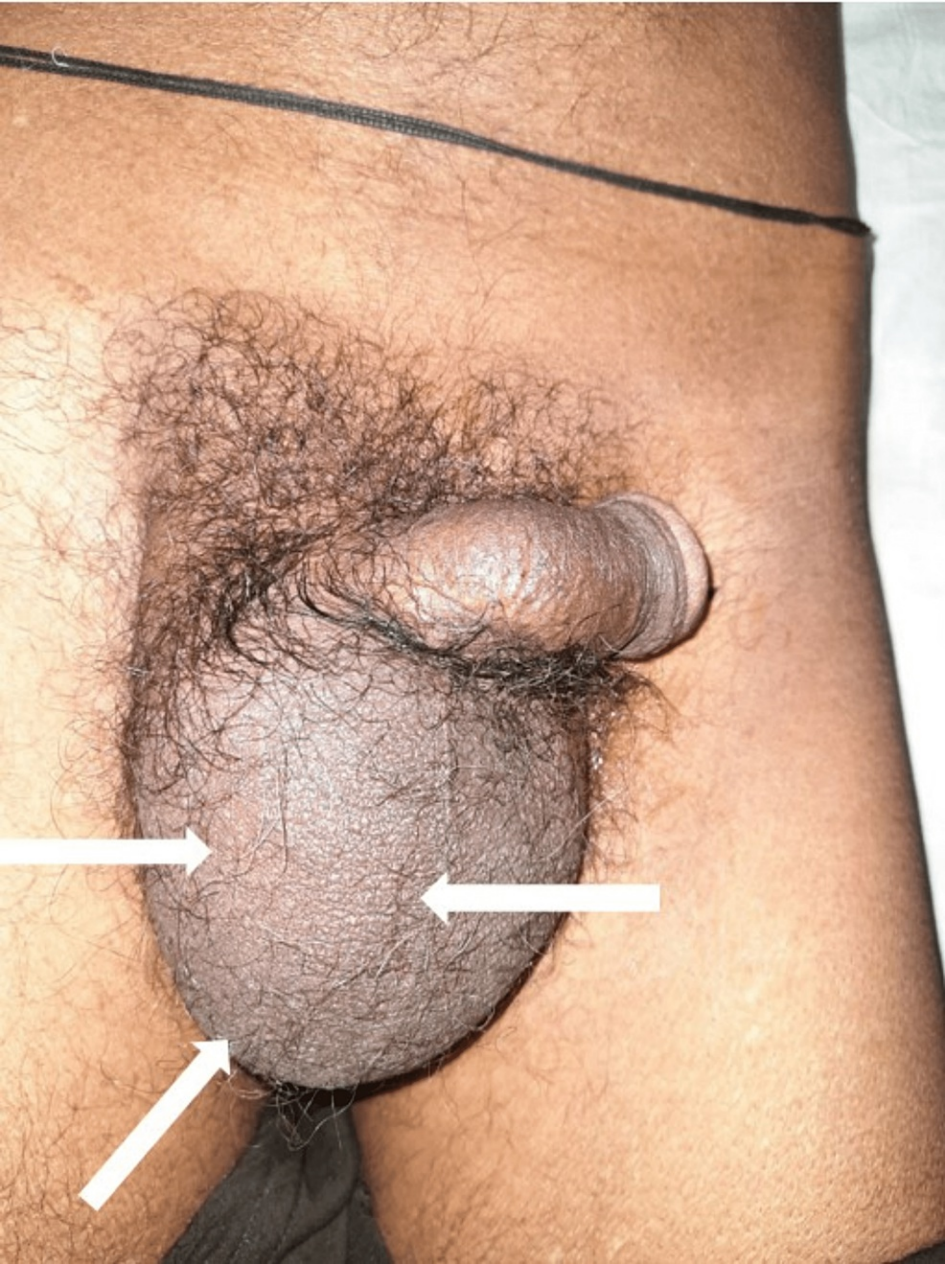
Clinical photograph showing right scrotal swelling with loss of skin rugosities

A 68-year-old male presented with right scrotal swelling gradually increasing in size for two and half months (Figure 1).

**Figure 2:**
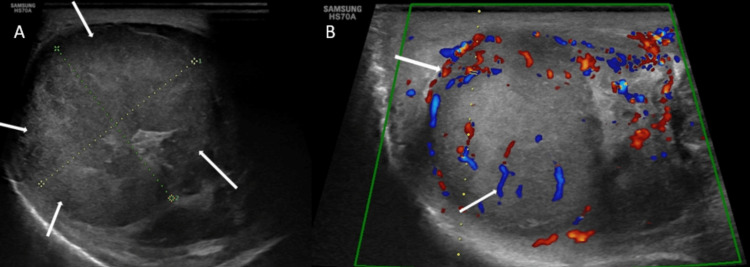
A: High-frequency ultrasound showing a heterogenous, predominantly hypoechoic mass in the right scrotal sac, replacing the right testis. B: On color Doppler, the mass was highly vascular.

The swelling was associated with pain and tenderness; the pain was dull and aching in nature. The patient also complained of pain in the abdomen and loss of weight. There was no history of trauma. On local examination, the skin over the swelling showed a loss of rugosities. There was no ulceration or fixation of the skin to underlying swelling. The swelling was hard, bulky, fully mobile, and measured 8.1 x 5.1 x 4.0 cm. The right testis could not be palpated separately from the lesion. The spermatic cord was palpated normally and showed no tenderness. The abdomen was soft and non-tender. The clinical impression was a right testicular mass. On inguinoscrotal high-frequency ultrasound, the right testis showed a heterogeneous, predominantly hypoechoic mass measuring 6.8 x 4.2 x 4.1 cm (volume 60 cc) (Figure 2A). No calcification was present within. The lesion was hypervascular on color Doppler (Figure 2B).

**Figure 3:**
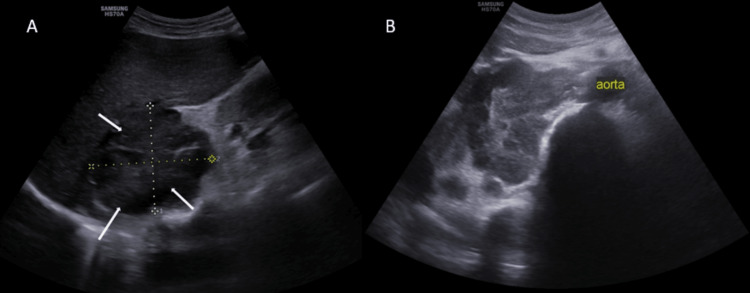
A: Abdominal ultrasound showing right adrenal mass. B: Abdominal ultrasound showing right para-aortic lymph nodal mass.

Multiple sub-centimetric lymph nodes were present in the inguinal region bilaterally. Abdominal ultrasound showed a heterogeneous mass of size 6.0 x 7.2 cm in the supra-renal area on the right side (Figure 3A). There were multiple enlarged and conglomerated lymph nodes in the pre-aortic, para-aortic, and renal hilum regions bilaterally (Figure 3B).

**Figure 4:**
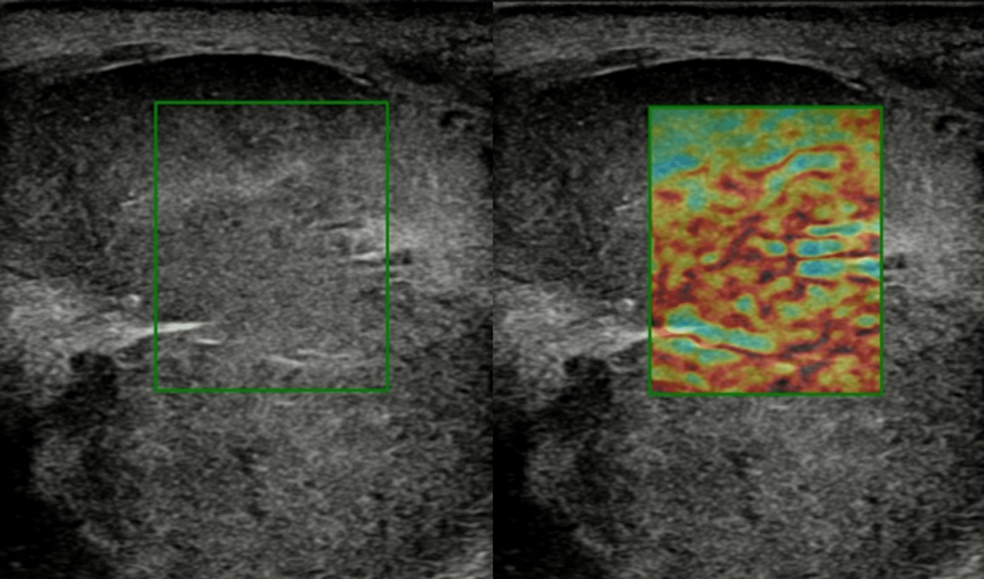
Shear wave elastography of the testes showing a stiff mass replacing the right testis

These lymph nodes were extending along the aorta up to the aortic bifurcation. Elastography of the testes demonstrated a hard mass replacing the right testis (Figure 4).

**Figure 5:**
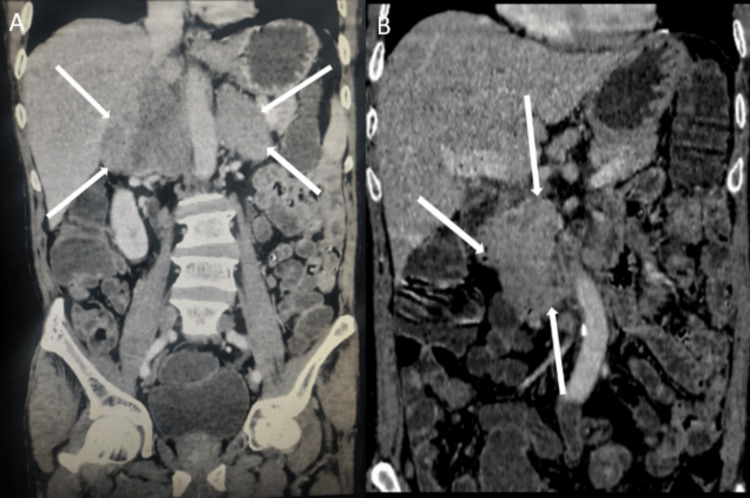
A: Coronal section of contrast-enhanced CT of the abdomen and pelvis showing bilateral adrenal metastasis. B: Coronal section of contrast-enhanced CT showing a hypodense omental metastatic mass in the subhepatic area.

Abdominal contrast-enhanced computed tomography (CECT) scan showed a hypodense lesion measuring 9.4 x 3.4 cm in the right adrenal gland, without post-contrast enhancement, suggesting metastasis. The lesion was extending into segment VI of the liver and pushing the IVC and right renal vein anteriorly. The lesion also encased the right renal artery. A similar morphology lesion was also noted in the left supra-renal gland, measuring 6.5 x 4.0 cm (Figure 5A). A hypodense omental lesion measuring 1.7 x 1.7 cm was noted in the subhepatic region, representing omental metastasis (Figure 5B).

**Figure 6:**
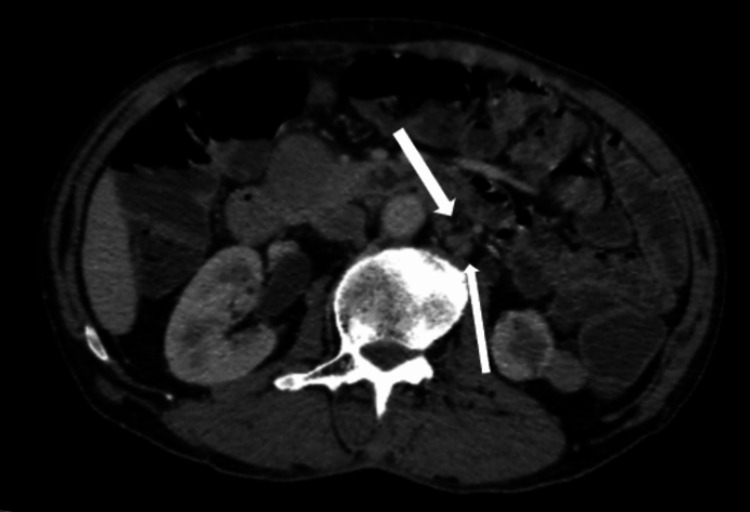
Axial section of contrast-enhanced CT abdomen showing multiple enlarged lymph nodes in the pre and para-aortic region.

Multiple lymph nodes were enlarged in the preaortic, para-aortic, right iliac fossa, left iliac fossa, and bilateral inguinal regions (Figure 6).

**Figure 7:**
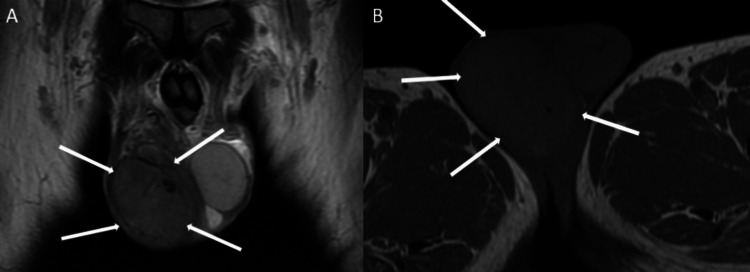
A: Coronal T2-weighted MRI section of the scrotum; the right testis is showing a hypointense mass completely occupying the testis. B: Axial T1-weighted MRI section of the scrotum; the right testis is showing an iso-intense mass completely occupying it.

MRI of the testes showed a hypointense lesion on the T2-weighted image completely replacing the right testis (Figure 7A). On the T1-weighted image, the lesion was isointense (Figure 7B).

**Figure 8:**
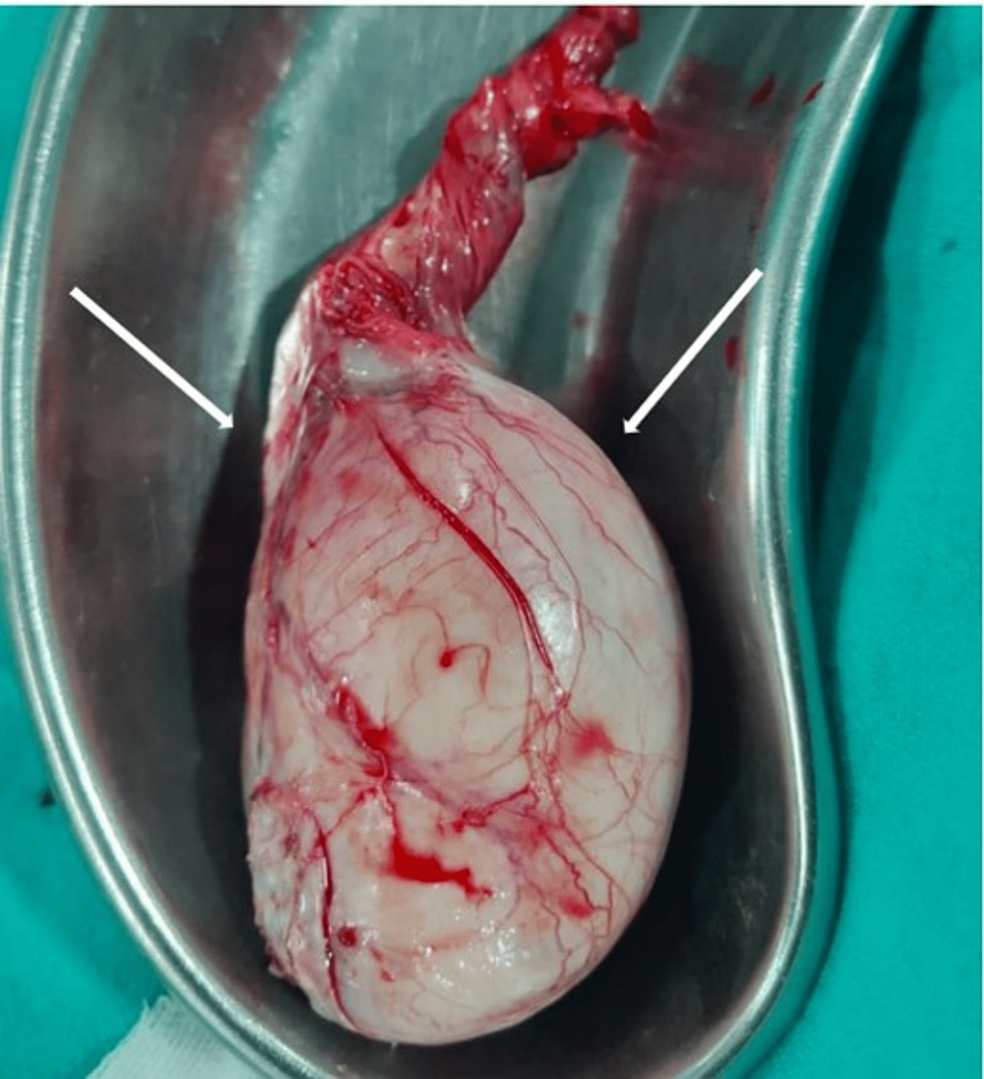
Gross specimen of the right high inguinal orchidectomy; the right testis is enlarged in size with increased vascularity.

The mass did not involve the tunica albuginea, scrotal wall, or epididymis. Tumor markers like alpha-fetoprotein were normal (<5 ng/dl), however, serum lactate dehydrogenase (LDH) was raised (552 IU/L). The patient underwent an adrenal gland biopsy and right high orchidectomy (Figure 8). On immunohistochemistry, CD20 and MUM1 turned out to be positive, suggestive of a high-grade B-cell lymphoma. Following this, the treatment was continued with systemic chemotherapy following established chemotherapy protocols for lymphoma. A combination of the rituximab, cyclophosphamide, doxorubicin, vincristine, and prednisone (R-CHOP) regimen was administered.

**Figure 9:**
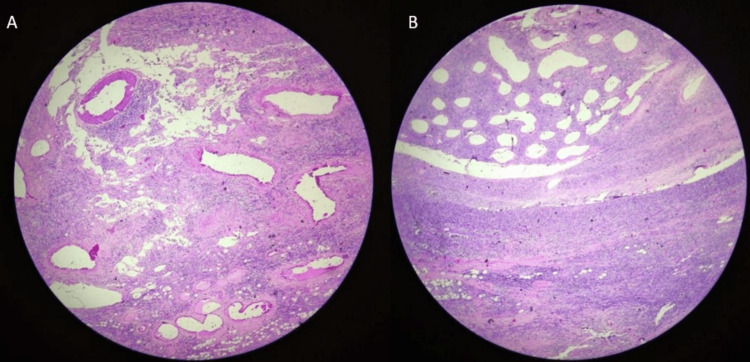
A: Histopathology of adrenal mass consistent with aggressive non-Hodgkin’s lymphoma; B: Histopathology of testicular mass consistent with aggressive non-Hodgkin’s lymphoma

The diagnosis of highly aggressive Non-Hodgkin’s lymphoma was confirmed on histopathology (Figures 9A, 9B).

## Discussion

Lymphomas are classified into two primary categories, namely, non-Hodgkin's lymphoma (NHL) and Hodgkin's lymphoma (HL). NHL is more common than HL and accounts for 85% of all lymphomas [5]. Among non-Hodgkin's lymphoma (NHL), diffuse large B-cell lymphoma (DLBCL) stands as the most prevalent subtype [6]. Extranodal involvement in lymphomas can occur in various locations such as the lung, omentum, peritoneum, genitourinary tract, bone, central nervous system, and bilateral adrenal glands [7]. Inguinoscrotal ultrasound is the primary modality for the evaluation of testicular masses. Scrotal ultrasound exhibits a well-defined heterogeneous predominantly hypoechoic mass, difficult to distinguish from germ cell neoplasms [8]. On color Doppler, the primary testicular lymphomas are hypervascular, irrespective of their size [9]. Malignant testicular tumors are stiff on elastography [10]. The important indicators on MRI that point toward the diagnosis of testicular carcinoma include predominantly low signal intensity or inhomogeneous variable signal intensity masses on T2- weighted images [11]. The sensitivity of MRI in detecting malignant testicular tumors is 100% while the specificity is 87.5%. The positive predictive value stands at 96.5% and the negative predictive value at 100%. Overall, the MRI demonstrates an accuracy of 96.4% [12]. Contrast-enhanced computed tomography of the head, abdomen, chest, and pelvis is done for staging and determining extranodal involvement. Consideration of age group and multifocal involvement along with an assessment of serum human chorionic gonadotropin (HCG), alpha-fetoprotein, and lactate dehydrogenase levels is important to reach the diagnosis and to rule out germ cell tumors. Complete blood counts and peripheral smears are done to know the bone marrow involvement. Confirmative diagnosis is reached by inguinal orchiectomy and histopathology. As much as 25 % of cases of lymphoma have adrenal involvement at autopsy. Treatment mainly consists of chemotherapy [8]. Of all extranodal lymphomas, primary testicular lymphoma has the worst prognosis. Five-year survival rate ranges between 70-79 % [13].

Clemens JQ published a comparable case report on testicular lymphoma with bilateral adrenal metastasis, elaborating on the findings of ultrasound, computed tomography, PET-CT, and histopathology of the testicular lump. However, the report failed to exclude the potential for dual malignancy [8]. Biradar BP also presented a similar case report that emphasizes the characteristics observed across all fundamental diagnostic modalities, alongside clinical presentation and treatment details. Nevertheless, the report omitted the MRI features of testicular lymphoma [14].

## Conclusions

In a patient above 50 years of age presenting with scrotal swelling, a high level of suspicion should be maintained for testicular lymphomas. Ultrasound plays a primary role in diagnosing the tumor while computed tomography is useful for staging the tumor. Elastography and MRI further help in the accurate diagnosis of the tumor and help in the management of such cases.
